# Supplementary material for: A Pencil-Lead Immunosensor for the Rapid Electrochemical Measurement of Anti-Diphtheria Toxin Antibodies
Source: Biosensors (Basel). 2021 Nov 30;11(12):489. doi: 10.3390/bios11120489 (PMC8699316; doi:10.3390/bios11120489)
Supplement: Supplementary file 1 [file biosensors-11-00489-s001.zip › biosensors-1465982-SI.pdf]

biosensors

MDPI

Supplementary Material

A Pencil-Lead Immunosensor for the Rapid Electrochemical Measurement of Anti-Diphtheria Toxin Antibodies

Wilson A. Ameku [1], Vanessa N. Ataide [2], Eric T. Costa [2], Larissa R. Gomes [1], Paloma Napoleão-Pêgo [1], David W. Provance, Jr. [1], Thiago R. L. C. Paixão [2,3], Maiara O. Salles [4] and Salvatore G. De-Simone [1,5,6,*]

[1] Oswaldo Cruz Foundation (FIOCRUZ), Center for Technological Development in Health (CDTS)/National Institute of Science and Technology for Innovation in Neglected Populations Diseases (INCT-IDPN), Rio de Janeiro, RJ 21040-900, Brazil; akira.ameku@gmail.com (W.A.A.); larissa.gomes@cdts.fiocruz.br (L.R.G.); ppego@cdts.fiocruz.br (P.N.-P.); bill.provance@cdts.fiocruz.br (D.W.P.J.)

[2] Department of Fundamental Chemistry, University of São Paulo, Institute of Chemistry, São Paulo, SP 05508-000, Brazil; vanneiva@usp.br (V.N.A.); eric.costa@usp.br (E.T.C.); trlcp@iq.usp.br (T.R.L.C.P.)

[3] National Institute of Bioanalytical Science and Technology, Campinas, SP 13084-971, Brazil

[4] Institute of Chemistry, Federal University of Rio de Janeiro, Rio de Janeiro, RJ 21941-909, Brazil; maiara@iq.ufrj.br

[5] Cellular and Molecular Department, Biology Institute, Federal Fluminense University, Niterói, RJ 24020-141, Brazil; dsimone@cdts.fiocruz.br

[6] Epidemiology and Molecular Systematics Laboratory, Oswaldo Cruz Institute, FIOCRUZ, Rio de Janeiro, RJ 21040-900, Brazil; dsimone@cdts.fiocruz.br

* Correspondence: dsimone@cdts.fiocruz.br; Tel.: +55-21386-58183

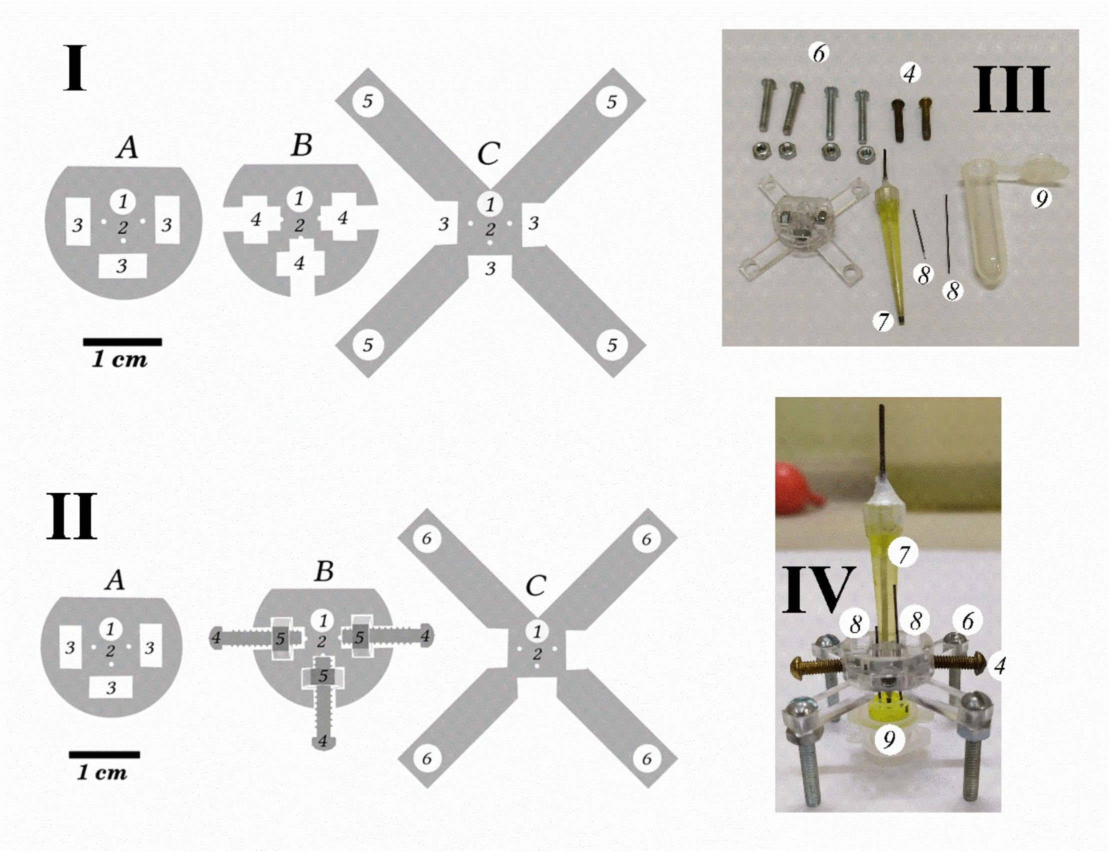

Figure S1. (**I**) Drawing the electrode holder. They were made stacking three sheets of PMMA where A, B, and C are the top view, middle and bottom layers, respectively. 1—hole for the reference electrode. 2—Three holes to add PLEs electrodes. 3—places for nuts, 4 -places for nuts and screws for electrode hold, 5—places for screws to adjust the holder height. (**II**) Top view of the disassembled electrode holder. A, B and C are the top, middle and bottom layers, respectively. 1 - hole

for the reference electrode, 2 - Three holes to place the PLEs. 3—places for nuts. 4 - Screws to hold the PLEs. 5 - Nuts. 6 - Places to add the screws to height adjust. (III and IV) Photo of the (III) dis- and (IV) assembled holder. 4—Screws to hold the PLEs. 6—Screws to height adjust. 7—Reference electrode. 8—PLEs 9—microcentrifuge tube or its cap.

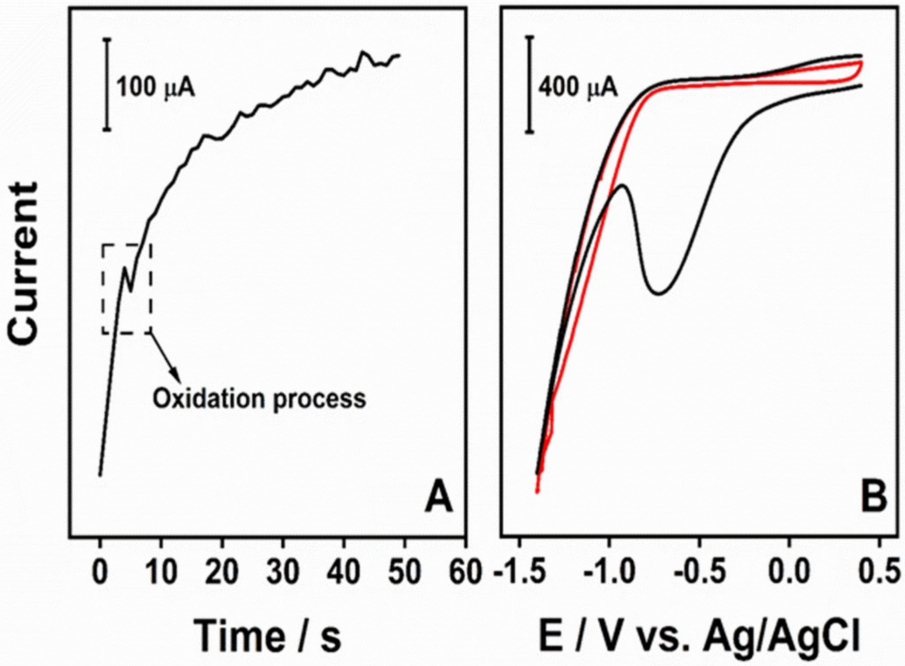

Figure S2. Oxidation of the working surface of the PLE. (**A**) Chronoamperogram obtained by the application of +2V for 50 s in a vigorously stirred solution of 0.1M PBS (pH 7.4) using a bare PLE as the working electrode. The initial oxidation in boxed. (**B**) Cyclic voltagram of the first (black) and second (red) cycle showing the improvement by the oxidization of PLE as working electrode. The reference and auxiliary electrodes were Ag/AgCl (KCl 3 mol L^{-1}) and PLE, respectively.

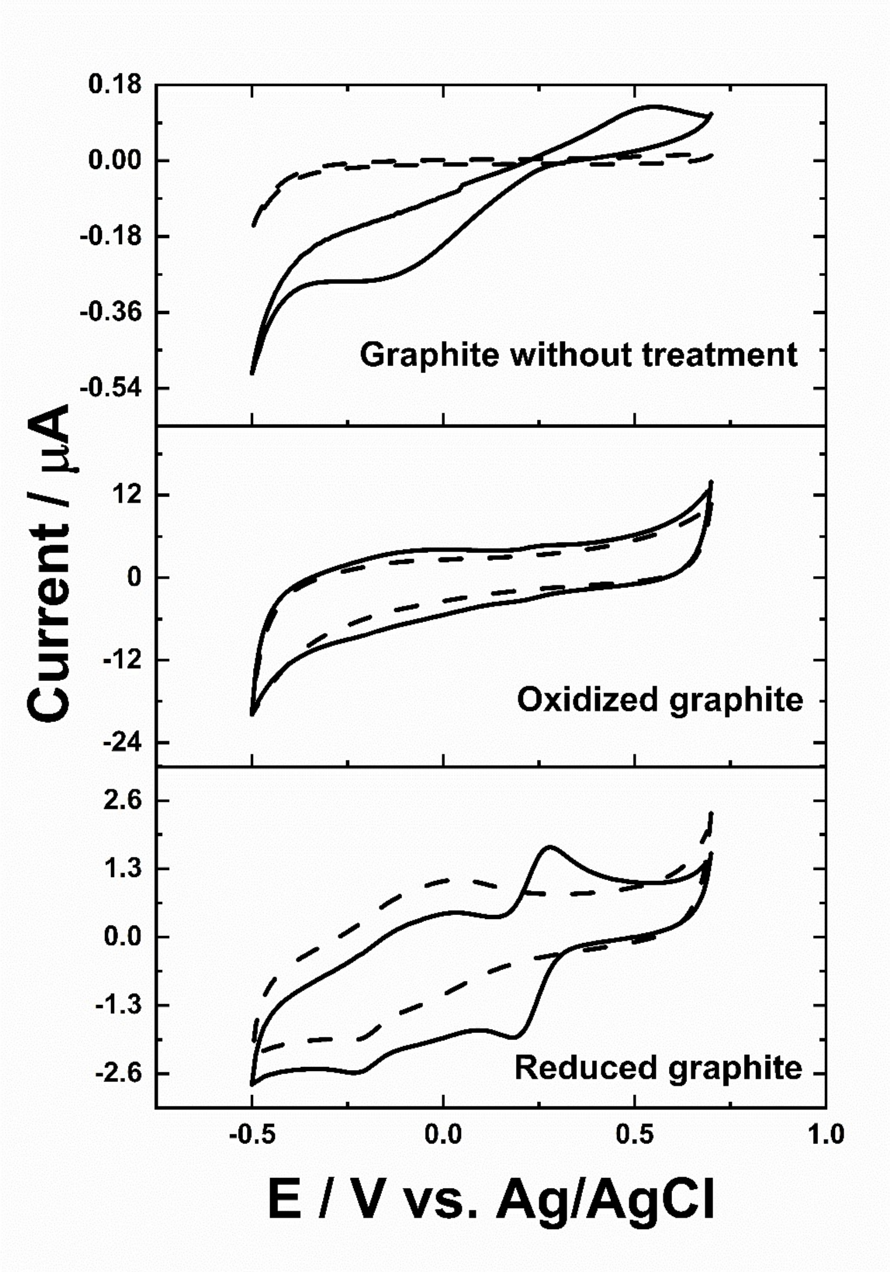

Figure S3. Influence of surface modification on the performance of PLE. Cyclic voltammetry (CV) recorded in 0.1 mol L^{-1} PBS (pH 7.4) alone (dashed line) or with 3 mM $Fe[(CN)_6]^{4-}$ (solid line) for unmodified PLE (A), oxidized graphite (B) and reduced graphite (C). Before and after electrochemical treatment (reduced graphite), the peak separation decreased from 670 mV to 90 mV. The peak intensity increased 7-fold featuring an electron transfer improvement. The oxidized graphite presented a large capacitive current and poor electron transfer property demonstrated by less defined peaks. In all cases, the scan rate was 100 mV/sec with bare PLE and Ag/AgCl (KCl 3 mol L^{-1}) as auxiliary and reference electrodes, respectively.

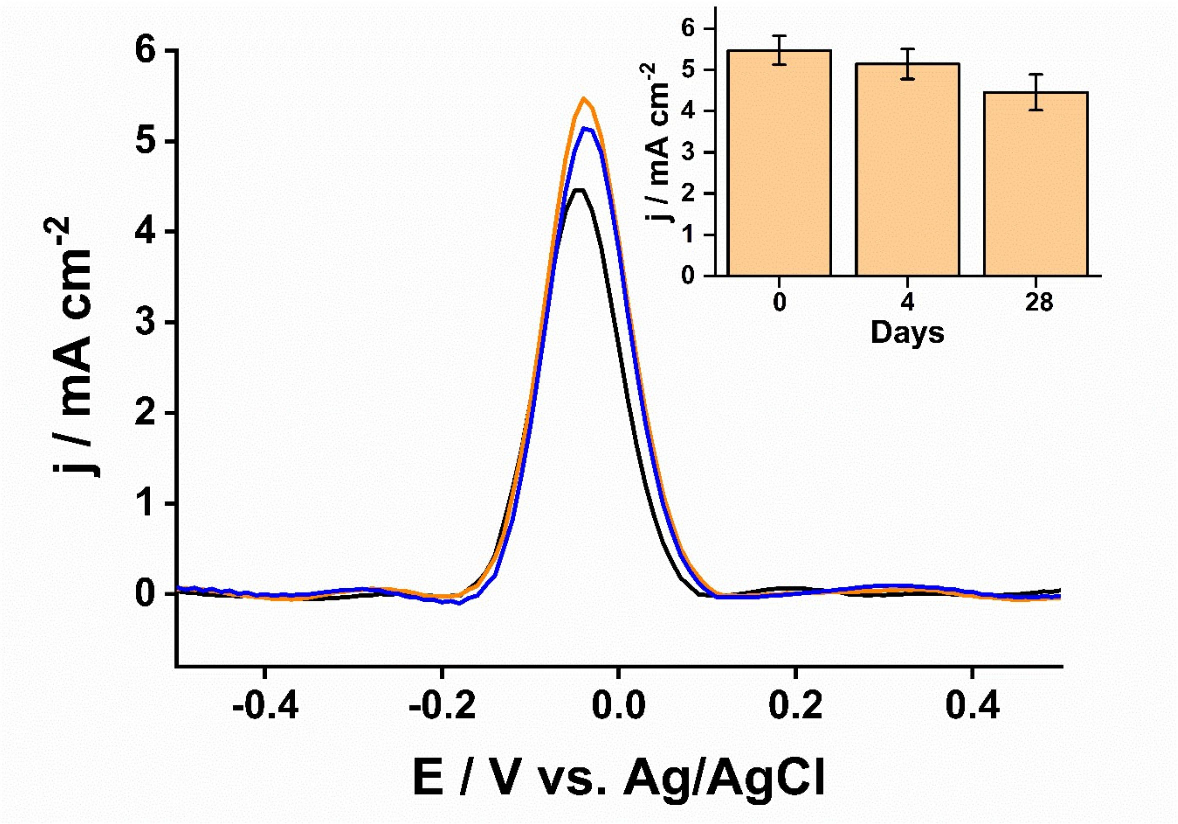

Figure S4. SWVs were recorded in 5 mmol L^{-1} of diPho-HQ prepared in 0.1 mol L^{-1} Tris-HCl/0.02 mol L^{-1} $MgCl_2$ solution (pH 9.8) after incubating PLE/biEP/BSA in 10^{-4} IU mL^{-1} IgG solution to evaluate the device's reproducibility (orange line, $n = 5$) and stability after 4 (blue line, $n = 3$) and 28 (black line, $n = 3$) days of storage at 4 °C. The experiments were performed using different electrodes; in the case of the reproducibility test, they were prepared in the same manner on different days. The inset bar graph is SWV-current variations with their standard deviations recorded after different days of storage.

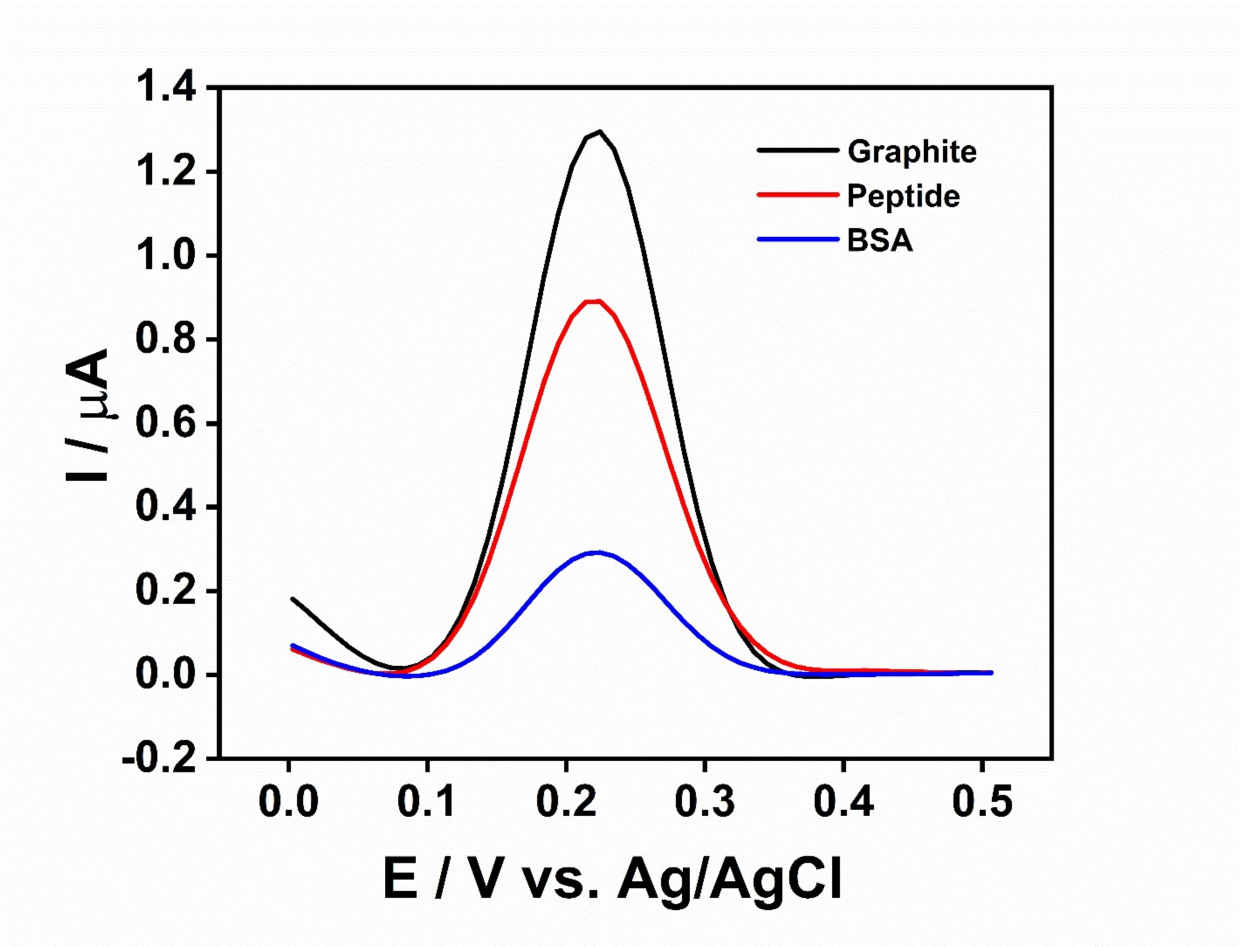

Figure S5. SWVs were recorded in a mixture of 5 mmol L^{-1} $Fe(CN)_6^{3-/4-}$ in 0.1 mol L^{-1} KCl in each stage of the PLE surface modification. Bare PLE (black line), PLE/biEP (red line), and PLE/biEP/BSA (blue line). SWV parameters: amplitude of 10 mV, a step of 10 mV, and frequency of 6.3 Hz.

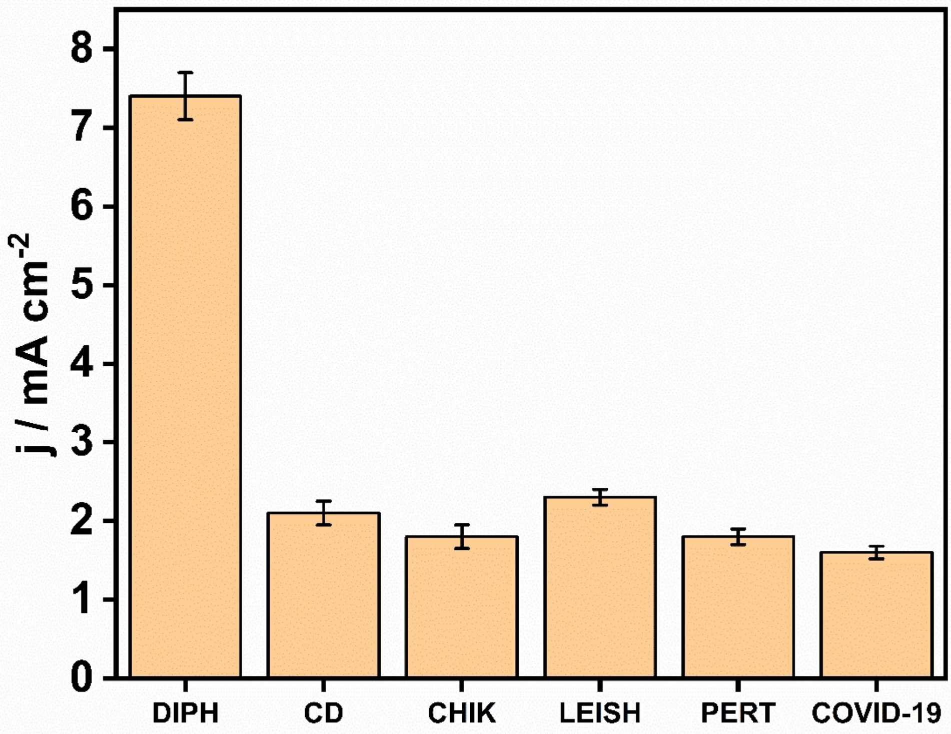

Figure S6. Absence of cross-reactivity to antibodies against other pathogens. PLEs were used to assay serum from patients with Chagas disease (CD; *Typanosoma cruzi*), Chikungunya (CHIK), Leishmaniosis (LEISH), Pertussis (PERT), and COVID-19 (SARS-CoV-2). The median signal from SWVs is presented with the standard deviation from assays performed in triplicate.
